# Diagnosing Emerging Fungal Threats: A One Health Perspective

**DOI:** 10.3389/fgene.2018.00376

**Published:** 2018-09-11

**Authors:** Pria N. Ghosh, Matthew C. Fisher, Kieran A. Bates

**Affiliations:** ^1^Department of Infectious Disease Epidemiology, Imperial College London, London, United Kingdom; ^2^Unit for Environmental Sciences and Management, North-West University, Potchefstroom, South Africa; ^3^Institute of Zoology, Zoological Society of London, London, United Kingdom

**Keywords:** emerging fungal pathogens, mycoses, one health, disease ecology, epidemiology, diagnostics, genomics, next generation sequencing

## Abstract

Emerging fungal pathogens are a growing threat to global health, ecosystems, food security, and the world economy. Over the last century, environmental change and globalized transport, twinned with the increasing application of antifungal chemical drugs have led to increases in outbreaks of fungal diseases with sometimes catastrophic effects. In order to tackle contemporary epidemics and predemic threats, there is a pressing need for a unified approach in identification and monitoring of fungal pathogens. In this paper, we discuss current high throughput technologies, as well as new platforms capable of combining diverse data types to inform practical epidemiological strategies with a focus on emerging fungal pathogens of wildlife.

## Introduction

Emerging fungal pathogens (EFPs) present an increasing threat to public health, food security, and ecosystems. Despite the risk that mycoses pose, a review of United Kingdom investment by philanthropic and public funding institutions found that between 1997 and 2010, research relating to mycoses was the focus of under 3% of funded studies, or an underwhelming 1.9% of the financial investment in all infectious disease research (Brown et al., 2012a,b; Fisher et al., 2012; Head et al., 2014). Poor investment in surveillance, diagnosis, and reporting makes assessing the true burden of fungal pathogens difficult (Brown et al., 2012a; Bongomin et al., 2017), but mycoses exert heavy morbidity. Over a billion people are directly affected by mycoses globally, 150 million of whom have a serious or life threatening infection (Brown et al., 2012a,b; Head et al., 2014; Gow and Netea, 2016; Bongomin et al., 2017). Furthermore, reports of EFPs are rising worldwide (Brandt and Park, 2013; Vallabhaneni et al., 2016; Benedict et al., 2017), driven through a combination of geographic expansion of pathogenic fungi, climate change, modified land use and increased use of immunosuppressive and antifungal drugs (Brandt and Park, 2013; Benedict et al., 2017). More overlooked is the spread of disease in wildlife where a broad range of species have experienced extirpations or even extinctions due to EFPs (Fisher et al., 2012).

It is estimated that up to 98% of all fungi remain unclassified (Hawksworth and Lucking, 2017) and there is a shortage of classically trained mycologists with the expertise to isolate and characterize novel species (Steinbach et al., 2003; Kozel and Wickes, 2014). Even the challenge of identifying a pathogenic fungus to species level may not be sufficient – hybridisation and local adaptation can lead to cryptic speciation and the evolution of intraspecies lineages which may vary in their level of virulence, such as in the case of *Batrachochytrium dendrobatidis*, the amphibian skin pathogen (Farrer et al., 2011). Many fungi cannot easily be grown under lab conditions, and culturing is time consuming and requires specialist training (Irinyi et al., 2016). Therefore, there is a need for diagnostics that can be widely applied by epidemiologists lacking traditional fungal typing skills. While methods for diagnosing human mycoses have been well reviewed in the literature (Kozel and Wickes, 2014), there has been little focus on diagnosis in wild animals. This is surprising given that over 60% of emerging infectious diseases in humans are of zoonotic origin, and the impact EFPs have on biodiversity worldwide (Jones et al., 2008). We discuss current and prospective methods available to researchers and personnel working in the field of wildlife diseases with a particular emphasis on rapid, high throughput diagnostics suited to disease outbreak scenarios.

## MinIon and SmidgIon

In 2014 Oxford Nanopore Technologies unveiled the MinIon, the first and currently only portable real-time DNA and RNA sequencer (Oxford Nanopore Technologies, Oxford, United Kingdom). Weighing just under 100 g, the MinIon is able to generate 10–20 Gb of DNA sequence data for the relatively cheap price of $1000 per starter pack (pricing as of June 2018). The data is immediately accessible and long reads make it ideal for sequencing the large, complex genomes of fungi. This technology was tested in a resource limited setting in 2015, when researchers rapidly generated sequence data for Ebola PCR amplicons in Guinea, (Quick et al., 2016) and again in the Americas when MinIon was used to track the spread of the Zika virus (Quick et al., 2017). In a clinical setting during the United Kingdom’s largest outbreak of *Candida auris*, MinIon’s rapid processing time enabled researchers to quickly identify multiple antifungal resistance alleles in some of the patient samples, and demonstrate the outbreak’s Asian origin (Rhodes et al., 2018). Now, Oxford Nanopore is developing the SmidgIon. The aim is to further simplify preparation requirements and shrink the technology, enabling a 10 min library preparation and a sequencer that can be plugged into a smartphone^1^.

MinIon’s portability is its key advantage as well as, like other sequencing technologies, requiring no prior knowledge of the pathogen genome, enabling a faster response time and the detection of ‘unknown unknowns’ (Juul et al., 2015). The biggest barriers to widespread adoption of MinIon for wildlife epidemics are cost and expertise in interpreting results and genome assembly. However, as the technology becomes more widely and routinely used, it is likely that these barriers will be reduced.

It is not currently possible to culture many fungi *in vitro* (Jeewon and Hyde, 2007), but DNA sequencing also has the potential to address this issue. The Known Media Database (KOMODO) is a novel web-based platform that collates information on over 20,000 organism-media pairings relating to approximately 18,000 bacteria and archaea species and over 3,000 media variants. The database predicts a suitable culture medium for an archaea or bacteria given the 16S rDNA sequence (Oberhardt et al., 2015). A similar database for fungi could reduce time required to isolate an unknown fungus for the first time.

## Loop Mediated Isothermal Amplification (LAMP)

Polymerase Chain Reaction based applications are still often viewed as the gold standard for rapid pathogen diagnosis, but require an expensive and cumbersome thermocycler, refrigeration of costly reagents, and trained personnel.

Loop Mediated Isothermal Amplification is a one-step isothermal amplification reaction involving a number of target specific primers (Mori and Notomi, 2009). No thermocycler is required, so it is substantially cheaper and more portable than traditional PCR based techniques, and is suitable for use in a field setting (Mori and Notomi, 2009). LAMP is highly specific, at least as sensitive as conventional PCR, and is rapid (a 10^9^ yield in DNA copy achievable in under an hour) (Notomi et al., 2000; Niessen, 2015). Real-time quantification is possible, for example by using a photometer to detect changes in turbidity that occurs through the generation of a reaction by-product (insoluble magnesium pyrophosphate) (Mori and Notomi, 2009).

There are two major, but not insurmountable, drawbacks to LAMP as a diagnostic. Firstly, although the assay itself is simple to operate, the primer design requires expertise, and knowledge of the target pathogen genome. Secondly, the primers must be kept cool in the field. Despite this, LAMP is still much more flexible than PCR based approaches and its speed, sensitivity, simplicity, low cost and portability make it an ideal candidate method for use in future and ongoing wildlife epidemics.

## Chemical Characterization

Analysis of the chemical composition of microbial cells for taxonomic identification is routine in microbiological laboratories. Common approaches utilize mass spectrometry methods that ionize chemical compounds into charged molecules and measure their mass to charge (*m/z*) ratio.

The *m/z* ratio is determined by measuring the mass and charge of a chemical feature when it is detected by a mass spectrometer. Each microbe has a characteristic mass spectrum enabling identification by comparison to databases of known microbe spectra (Singhal et al., 2015). Strain level identification is possible, for example of pathogenic fungi such as *Candida sp.* (Qian et al., 2008; Pulcrano et al., 2012; Aslani et al., 2018).

A frequently applied mass spectrometry based diagnostic for pathogen detection is matrix assisted laser desorption ionization time of flight mass spectrometry (MALDI-ToF MS) which detects microbe specific proteins. MALDI-ToF MS is especially popular owing to its congruence with DNA sequencing methods (Marklein et al., 2009; Thouvenot et al., 2018) and low cost (Tran et al., 2015).

While MALDI-ToF offers rapid results in a laboratory setting, its application as a diagnostic for outbreaks of unknown fungal pathogens is limited since microbial culture and reference spectra are required. Direct analysis of microbes from biological samples have yielded significant improvements in diagnosis time (Lockwood et al., 2016), though in some cases with reduced sensitivity (Singhal et al., 2015; Íñigo et al., 2016; Zboromyrska et al., 2016). Culture independent methods also often require additional sample preparation to remove cellular debris (Íñigo et al., 2016). Finally, while the test time is rapid and the analysis cost per sample is cheap, initial equipment acquisition is expensive (Tran et al., 2015).

More recently other mass spectrometry methods have been developed that hold potential as fungal diagnostics. Rapid evaporative ionization mass spectroscopy (REIMS) identifies microbes based on their lipid content and was able to identify cultured pathogenic *Candida* species with 98% accuracy (Strittmatter et al., 2014). Infrared spectroscopy (Quintelas et al., 2017) and Raman spectroscopy (Lorenz et al., 2017) are also promising and require minimal sample preparation. Refinements for direct sample analysis and high sensitivity in discriminating pathogens in complex microbial communities would benefit the diagnosis of fungal pathogen outbreaks.

## Lateral Flow Assays and Biosensors

The Lateral Flow Assay (LFA) is widely used and usually comes in a portable dipstick format. LFAs are normally designed to detect antigens or host-produced antibodies specific to a pathogen of interest and are often used to generate rapid test results in human clinical settings (Marot-Leblond et al., 2004; Thornton, 2008; Kozel and Wickes, 2014). LFAs have also been developed to test for several wildlife diseases such as amphibian chytridiomycosis (Dillon et al., 2017) and mammalian sylvatic plague (Abbott et al., 2014). Recently, LFA based diagnostics have diversified to include nucleic acid detection. Detection of a pathogen is indicated by a color change as the target DNA or antigen is bound by the LFA antibody or probe. The intensity of color change is proportional to the amount of target present, enabling the development of semi-quantitative tests using smartphone devices which have been used in a range of applications including detection of fungal toxins and antifungal resistance alleles (Lee et al., 2013). LFAs are particularly attractive diagnostics for wildlife disease outbreaks due to ease of use by non-specialists, minimally invasive sample collection, portability and rapid result generation (∼10–30 min) (Kozel and Wickes, 2014). The low cost of LFAs makes them an ideal front line diagnostic. LFA drawbacks include long development time, false positives and lower sensitivity compared to other methods (Kozel and Burnham-Marusich, 2017). It is therefore generally recommended that test results should be corroborated using more sensitive lab-based diagnostics.

Microfluidic biosensors are also increasingly being applied for point of care diagnosis of human pathogens, and could be excellent candidates for application to wildlife epidemiology. “Biosensor” applies to a wide range of devices which are able to identify and quantify the amount of a target species or biomolecule (Prakash et al., 2012). Microfluidic devices are often chip based, and channel samples through a series of miniaturized components including those for sample preparation, target detection and data processing (Jayamohan et al., 2013; Pandey et al., 2017). An ideal microfluidic biosensor diagnostic should be cheap [for example, it is possible to make microfluidic devices from wax and paper (Nilghaz et al., 2016)], easy to use by a non-specialist, fast, and utilize non-invasive sample collection. This has been demonstrated for several human pathogens, including for *Plasmodium falciparum* (Fraser et al., 2018) and *Escherichia coli* (Altintas et al., 2018) but requires further development and validation for wildlife pathogens (Ray et al., 2017).

## Data Collection and Collation

New technology has enabled collection of greater quantities of field data. The question then follows – how to manage, interrogate and visualize it all? Wildlife epidemiological fieldwork often takes place in resource poor environments and under time sensitive conditions. It may be necessary to have multiple teams sampling in different places, requiring easily collatable, consistent sample collection and recording. EpiCollect (Aanensen et al., 2009) [and, more recently developed, EpiCollect+ (Aanensen et al., 2014)] is a novel open source data management platform, compatible with any smartphone. Multiple phones can be linked to a project, with geotagging capabilities. Users, regardless of location, can view, edit, analyze or download data with a smartphone. EpiCollect and EpiCollect+ are increasingly widely applied, including for: research into controlling schistosomiasis in Mozambique (Phillips et al., 2018); modeling malaria transmission patterns across four sub-Saharan African countries (Marshall et al., 2016); mapping the distribution of *B. dendrobatidis* in Taiwan (Fisher et al., 2018); and investigating HIV infections in Zimbabwe (Gregson et al., 2017). However so far, aside from its application to *B. dendrobatidis* mapping in Taiwan, EpiCollect has not been utilized for wildlife epidemiology or fungal pathogen research despite presenting an excellent opportunity to greatly increase the scope of epidemiological projects at minimal cost.

The question of how to visualize and present large volumes of complex data has also become pressing. Genomic data in particular can appear intimidating to non-experts, and yet in the context of an epizootic it is important for a wide range of personnel to be able to access and understand information on pathogen evolution and genomes (Argimón et al., 2016). Originally, sequence data from large distributed genotyping projects was databased, analyzed and distributed through online multilocus sequence typing (MLST) databases such as MLST^2^ and PubMLST^3^. Now, MLST databases are being superseded by the next generation of online genotyping databases that upload, map, analyze and display genome sequence data. In tools such as WGSA^4^, the sequence data can be directly uploaded via the web application along with metadata and interrogated via an interactive user-friendly interface. While the number of pathogens that can be analyzed in this manner is currently limited, it is only a matter of time before online databases for more, including key mycoses, are developed. Even if such databases have not been created, the phylogeographic output of pathogen genomic analyses can be displayed within the context of its metadata in flexible online resources such as Microreact^5^.

Microreact is not alone in presenting a novel way of approaching the management and visualization of genomic and epidemiological data. TransPhylo is an R package that computes the probability of an observed transmission tree for a pathogen given the phylogenetic tree (even under circumstances of incomplete sampling or an ongoing epidemic) (Didelot et al., 2017). TreeBreaker has been built for the evolutionary inference of phenotype distribution and has already been used to investigate the association between HIV genetic variation and human leukocyte antigens (Ansari and Didelot, 2016; Didelot et al., 2017). It is clear that fungal disease outbreak analysis increasingly occupies an informatic space where the development of open source toolkits that facilitate rapid analysis and dissemination of diverse data types are central to effective disease management.

## Conclusion

It is more urgent to monitor EFPs in wildlife now than ever before. In recent years mycoses have ravaged swathes of species, sometimes with catastrophic effects on biodiversity (Fisher et al., 2012; O’Hanlon et al., 2018). Globalization resulting in species redistribution and increased contact between hosts will inevitably enhance disease transmission, posing environmental and public health challenges on a worldwide scale. Specialists from a diverse range of fields including veterinary professionals, researchers and public health workers will need to work cooperatively and vigilantly to mitigate future disease outbreaks. Fundamental to any successful action plan will be the implementation of rapid and reliable diagnostics to identify the aetiological agent of disease, and subsequently monitor the spread of an epidemic.

**FIGURE 1:**
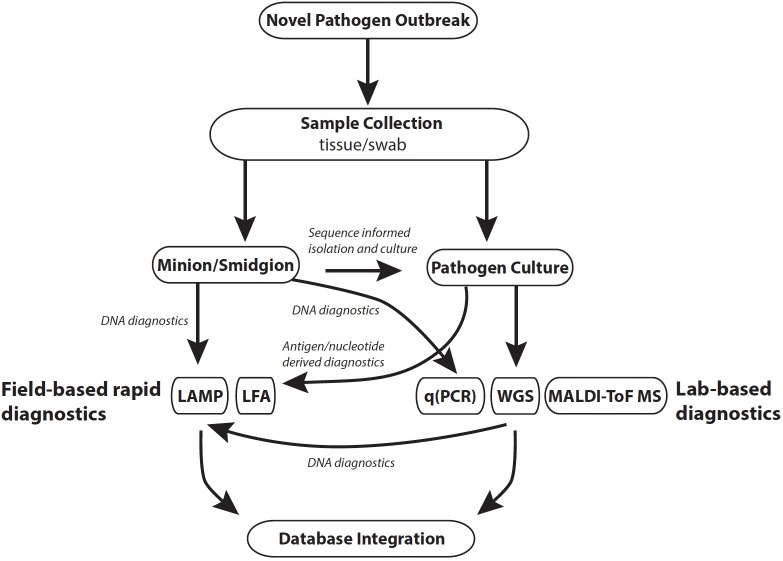
Outline of tools applicable to different stages of a pathogen outbreak. Database integration: R packages include TransPhylo and TreeBreaker; online databases include Microreact, EpiCollect, WGSA.net. Abbreviations: LAMP, loop mediated isothermal amplification; LFA, lateral flow assay; qPCR, quantitative polymerase chain reaction.

Effective monitoring of a disease outbreak will require a range of diagnostic methods generating diverse data that subsequently facilitates a holistic view of an epidemic, or epizootic (**Figure 1**). Diagnostics should be reproducible, straightforward to use, generate rapid results and be cost-effective. The choice of diagnostic is also dependent on the stage of an outbreak (**Table 1**). For example, in a scenario where the pathogen is unknown, common methods that require *a priori* reference data (e.g., reference spectra for mass spectrometry methods) would not be informative. In such instances, rapid and culture-free sequence based methods such as MinIon may be the first port of call in order to construct a reference genome (Farrer and Fisher, 2017; Langner et al., 2018). Once sequence-based pathogen identification is complete, it may be easier to isolate the fungus by inferring ideal culture conditions. At this point development of DNA based rapid diagnostics such as LAMP assays would be possible using the assembled whole genome sequence data. Once cultured, reference mass spectra in addition to development of LFAs for the pathogen could be developed. When a novel rapid diagnostic is validated to meet sensitivity and reproducibility requirements it can be rolled out to practitioners in the field. Effective modeling of disease dynamics and subsequent management strategies will be dependent on integrating multiple data types collected from different geographic regions as well as clinical microbiology laboratories. This is best implemented by uploading field data in real time from smart phone devices to online databases such as EpiCollect+. Once online, data can easily be disseminated for downstream analysis.

**Table 1:** Examples of proposed workflow applied to known emerging fungal pathogens of wildlife.

Pathogen (phylum)	Host	Emergence context	Diagnostic workflow
*Batrachochytrium dendrobatidis* (Chytridiomycota)	Amphibians	Worldwide emergence of a highly destructive and undescribed pathogen, identified by isolation from infected amphibians (Longcore et al., 1999; Skerratt et al., 2007; Olson et al., 2013; Berger et al., 2016).	*Pathogen culture* Isolation of undescribed pathogen *Field-based rapid diagnostics and Lab-based diagnostics* Ability to rapidly identify presence of novel pathogen required *Whole Genome Sequencing* Further analysis to identify evolutionary context of novel pathogen
*Fusarium* sp. (Ascomycota)	Sea turtles	Isolates of *Fusarium*, a known opportunistic pathogen, recovered globally in the wild from dead eggs of endangered sea turtles (Sarmiento-Ramírez et al., 2010, 2014).	*Pathogen culture* Isolation and identification of known pathogen in a novel host *Whole Genome Sequencing* Rapid diagnostics for the known pathogen already exist, so progress to WGS to investigate host jump drivers
*Ophidiomyces ophiodiicola* (Ascomycota)	Snakes	Severe declines of wild snake populations in Northeastern United States are associated with skin lesions. *O. ophiodiicola* has previously been isolated from captive snakes in Europe but has not been observed in the United States, or previously been associated with population declines (Allender et al., 2011; Clark et al., 2011; Lorch et al., 2016; Franklinos et al., 2017).	*MinIon/SmidgIon* Molecular identification of pathogen associated with skin lesions *Pathogen culture* Use sequencing data to inform pathogen culture and isolation *Field-based rapid diagnostics* Lab diagnostics for known pathogen *O. ophiodiicola* exist, so develop rapid diagnostics for field monitoring
*Aspergillus sydowii* (Ascomycota)	Coral	Isolates of *A. sydowii*, a known opportunistic pathogen, isolated from diseased coral showing evidence of aspergillosis driven mortality. Further investigation shows some coral to be asymptomatically infected (Smith et al., 1996; Nagelkerken et al., 1997; Soler-Hurtado et al., 2016)	*Pathogen culture* Identification of an opportunistic pathogen in a new host *Whole Genome Sequencing* Rapid diagnostics for the known pathogen already exist, so progress to WGS to investigate host jump drivers and variance in virulence
*Nosema* sp. (Microsporidia)	Bees	Multiple *Nosema* species found to be associated with colony collapses of various bee species. A potential driver, pathogen pollution via the importation and range expansion of commercial bumblebees and managed honeybees, exists but the role of Microsporidia in colony collapses is not equivocal (Ratnieks and Carreck, 2010; Paxton, 2015; Brown, 2017).	*MinIon/SmidgIon* Identification of multiple closely related pathogens associated with bee declines *Field-based rapid diagnostics and Lab-based diagnostics* Development of diagnostics able to distinguish between candidate pathogens required to enable ongoing monitoring


While diagnostics for fungal pathogens have come a long way, a great deal more could be done to improve preparation for future outbreaks. Funding more projects that characterize the huge unknown fungal diversity will provide better genomic and mass spectrometry databases that may enhance the way in which EFPs are first classified through identifying pathogen-associated characteristics using comparative approaches (Farrer and Fisher, 2017; Farrer et al., 2017). In this way, pathogens or pathogen hotspots can be identified alongside an assessment of where, and where not, the pathogen occurs (O’Hanlon et al., 2018). These data can then be integrated into a “predemic” assessment of the potential risk that a novel pathogen poses which, in turn, could inform trans-national organizations such as the World Organization for Animal Health (OIE) or the World Health Organization (WHO) that are able to coordinate biosecurity-relevant policy actions (Voyles et al., 2014). The development of standardized, cost-effective diagnostics combined with greater collaboration and data sharing will yield faster, more reliable information that is relevant to the rapid assessment and response to outbreaks. This will in turn enable more effective mitigation strategies to be implemented and in doing so help to stem future outbreaks of EFPs.

## Author Contributions

All authors wrote and researched the manuscript and contributed to editing.

## Conflict of Interest Statement

The authors declare that the research was conducted in the absence of any commercial or financial relationships that could be construed as a potential conflict of interest.

## Footnotes

1 https://nanoporetech.com/products/smidgion

2 http://www.mlst.net

3 http://www.pubmlst.org

4 http://www.wgsa.net

5 http://www.microreact.org
